# Deterministic and Probabilistic Analysis of a Simple Markov Model: How Different Could They Be?

**DOI:** 10.1007/s40258-021-00700-1

**Published:** 2022-01-20

**Authors:** Howard Thom

**Affiliations:** 1 Bristol Medical School: Population Health Sciences, University of Bristol, Bristol, UK; 2 Clifton Insight, Bristol, UK

## Background

Markov multistate models are among the most common models used for health economic evaluations [1]. These break diseases into a finite set of health states, divide a simulated cohort of patients amongst these states, and simulate their disease by allowing transitions between states over time. They are accepted by healthcare decision makers, including the National Institute for Health and Care Excellence (NICE) and Canadian Agency for Drugs and Technologies in Health (CADTH) [2, 3].

Evidence informing parameters of Markov models, such as state transition probabilities, costs and health-related utilities, is often limited [4]. Probabilistic analysis evaluates the model over a distribution of these parameters and bases decisions on the distribution of outputs; deterministic analysis evaluates the model at parameter means, giving only a single output for decision making. As a Markov model is a nonlinear function, the mean of a probabilistic analysis will not match the output of a deterministic analysis. This follows from the general statement that the expectation of a nonlinear function is not equal to the nonlinear function acting on the expectation, of which Jensen’s inequality on convex functions is a special case [5, 6]. This was demonstrated in practice in the NICE evaluation of rivaroxaban in coronary or peripheral artery disease. The deterministic incremental cost-effectiveness ratio (ICER) was GBP16,602 per quality adjusted life year (QALY) while the probabilistic ICER was GBP8138/QALY, a factor of two reduction [7]. Wilson made this point and argued that decision makers should use only probabilistic analysis [8]. Furthermore, official guidance from NICE and CADTH both recommend the use of probabilistic analysis as the base case [2, 3].

Despite publicly available examples, theoretical argument, and official guidance, deterministic analyses remain common. Even if the base-case analysis is probabilistic, modellers will often use deterministic sensitivity analyses. The usual response to criticism is “How different could they be?” In this simple simulation study, I will show that they could be very different.

## Exploratory Simulation Study

The simulation study is based on a comparison of two treatments using a discrete-time 3-state cohort Markov model with 6-month cycles and 5-year time horizon. States are either healthy, adverse event, and dead, and all patients begin in the healthy state. The adverse event state has lower health-related utility and higher costs than the healthy state. Treatment 1 is the reference while treatment 2 has lower mortality, more adverse events, and higher cost. In probabilistic analysis, utilities and costs are Normally distributed, probabilities of death and adverse event on treatment 1 are Gamma, and log odds ratios for treatment 2 relative to treatment 1 are Normal. For deterministic analysis, the means of each parameter are used while probabilistic analysis uses 1000 parameter samples.

In the simulation study 10,000 samples from Uniform distributions are used to define the distributions on probabilities and log odds ratios. I refer to each iteration of the simulation as a scenario to avoid confusion with simulations in the probabilistic analysis. Parameters of cost and utilities are held constant.

Under each scenario, cost-effectiveness summaries are calculated and compared between the deterministic and probabilistic analysis. Total costs and total QALYs, discounted at 3.5 % per year, are calculated. Incremental costs, QALYs, and net benefits (INB) at GBP20,000/QALY are calculated for treatment 2 relative to treatment 1, along with the ICER [2]. For probabilistic analysis, the probability that treatment 2 is cost effective at GBP20,000/QALY is calculated, which is a point of the cost-effectiveness acceptability curve (CEAC), as is expected value of perfect information (EVPI) [4].

Full details of the model and simulation are provided in the supplementary material. They were implemented in the R statistical programming language and code is in the supplementary material [9, 10].

## Results of Simulation Study

Comparison of conclusions under the two analyses are presented in detail in supplementary material. In brief, if treatment 1 is dominant under probabilistic analysis, it is also dominant under deterministic analysis in 99% of scenarios. If treatment 1 is only cost-effective and not dominant under probabilistic analysis, the deterministic analysis finds treatment 2 to be dominant or cost-effective in a small number of scenarios (1.8%). If treatment 2 is cost-effective but not dominant under probabilistic analysis, treatment 1 is dominant or cost-effective under deterministic analysis in a larger percentage of scenarios (6.9%).

**Fig. 1:**
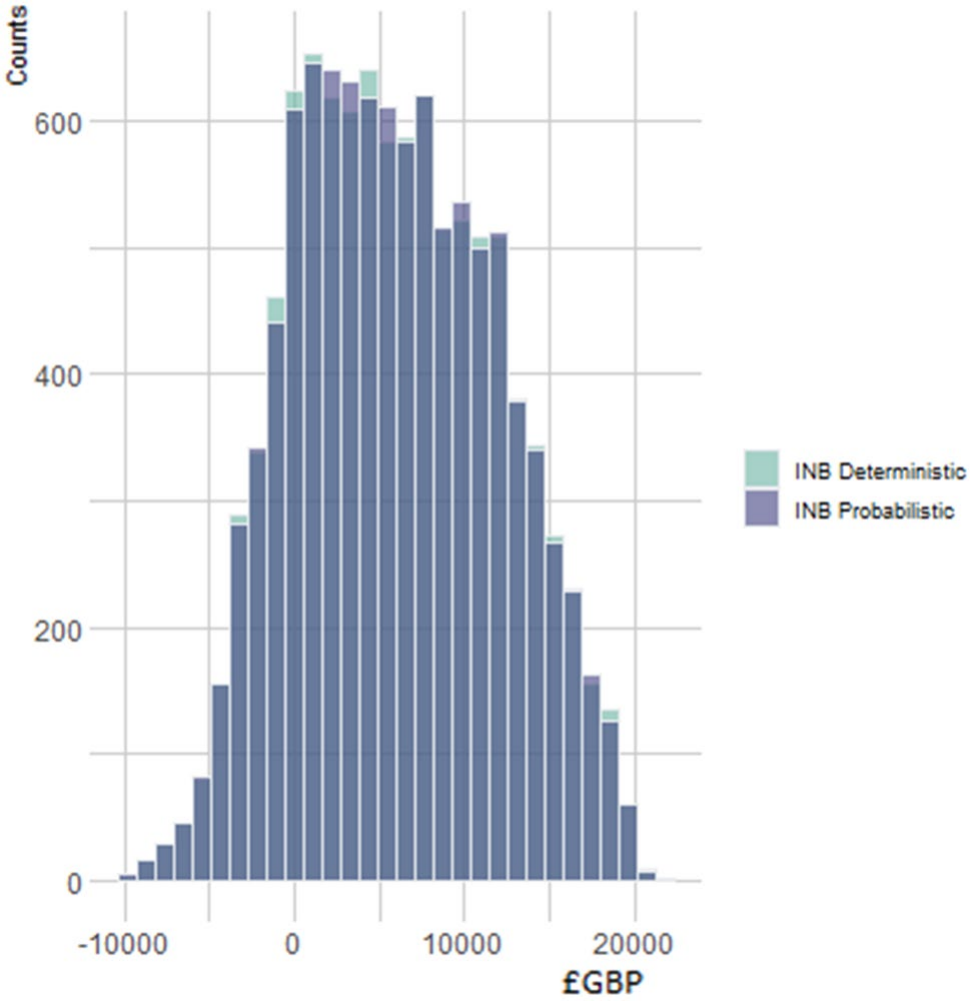
Histogram of incremental net benefit (INB) estimated by deterministic and probabilistic analysis across 10,000 scenarios*. *Across scenarios the mean and 95 % range of INB deterministic was GBP−2324.80 (− 12688.89, 9367.81), INB probabilistic was GBP6028.64 (− 4363.49, 17688.01), and probabilistic minus deterministic was GBP8353 (7955.72, 8731.12) giving definite evidence of a non-zero bias

Across scenarios, probabilistic INBs are similar to deterministic INBs (Fig. 1). The mean and 95% reference range under deterministic analysis was £6,021.82 (− 4359.20, 17707.67), under probabilistic £6,028.64 (− 4363.49, 17688.01), and probabilistic minus deterministic £6.82 (− 264.20, 243.35).

Results of the two worst case scenarios are tabulated in supplementary material. In a maximum ICER difference scenario, deterministic analysis found treatment 2 not cost-effective with ICER of £23,536/QALY while probabilistic analysis found treatment 2 cost-effective with an ICER of £19,661/QALY. In a maximum CEAC inconsistency scenario, deterministic and probabilistic analysis found treatment 2 not cost-effective with ICERs of £30,226/QALY and £26,382/QALY, respectively. However, the probabilistic analysis found 45% of simulations below £20,000/QALY, indicating substantial uncertainty.

## How Different Could They Be?

Our simple analysis of a 3-state Markov model found that results, and indeed conclusions, of deterministic and probabilistic analysis can be different. They disagreed on cost-effectiveness in 2–7% of scenarios and there were scenarios where the deterministic findings could be definitive (i.e., an ICER > £30,000/QALY) but the probabilistic analysis would indicate substantial uncertainty. The net benefit was found to be a more stable summary of cost-effectiveness than an ICER, so should be preferred in general.

Of course, there are limitations to this analysis. I considered only two treatment options and three health states and only a limited range of distributions for efficacy, cost and utility parameters. There was also no correlation between parameters. However, this analysis represents almost a minimum level of complexity for a Markov model. A greater range of model structures and parameter distributions could be explored in future simulation studies, and it is plausible that deterministic analyses will fail more frequently in more realistic cases.

## Recommendation

The recommendation is to avoid basing decision making on deterministic analysis when using a Markov model. This extends to any non-linear model such as a partitioned survival model or moderately complex decision tree. Even sensitivity analyses should not be deterministic as simply switching from probabilistic to deterministic can affect results and conclusions.

### Supplementary Information

Below is the link to the electronic supplementary material.

Supplementary file1 (DOCX 46 kb)

## Declarations

### Data and Code Availability

All data and code used in this paper are provided in the supplementary material.
