# Correction to: Deterministic and Probabilistic Analysis of a Simple Markov Model: How Different Could They Be?

**DOI:** 10.1007/s40258-022-00724-1

**Published:** 2022-03-18

**Authors:** Howard Thom

**Affiliations:** 1 Bristol Medical School: Population Health Sciences, University of Bristol, Bristol, UK; 2 Clifton Insight, Bristol, UK

## Correction to: Applied Health Economics and Health Policy 10.1007/s40258-021-00700-1

In the original online version of the article, there was an error in the R code that was identified by Dr. Sanchez Alvarez at Hoffmann-La Roche. The error was small but meant that treatment costs were being added to every cycle of the deterministic analysis, rather than only in the first cycle as in the probabilistic analysis. This substantially changes the cost estimates.

The headline result had been differences in conclusions when neither treatment was dominant, but these differences are much reduced. In these scenarios, if treatment 1 is cost-effective under probabilistic analysis the probability that treatment 2 is cost-effective but not dominant under deterministic changes from 54.9 to 1.6% after correcting the error (or if cost-effective or dominant changes from 55.1 to 1.8%). If treatment 2 is cost-effective under probabilistic the probability that treatment 1 is cost-effective but not dominant under deterministic changes from 94.3 to 6.3% (or if cost-effective or dominant changes from 94.9 to 6.9%). The difference in conclusions of 2–7% is still worrying but not sufficient to recommend probabilistic analysis in all cases.

Furthermore, the difference between the probabilistic and deterministic INBs change from £8353 (7955.72, 8731.12) to only £6.82 (− 264.20, 243.35). This leads to the conclusion that INBs are similar between probabilistic and deterministic analyses in this case.

The worst-case scenarios also changed substantially. The maximum ICER difference scenario had £160,861/QALY in deterministic and £19,661/QALY in probabilistic but is now £23,537 in deterministic vs £19,661/QALY (probabilistic unchanged). The maximum CEAC inconsistency was an ICER of £32,705/QALY in deterministic but a probabilistic probability of being cost-effective 1.00. This is now a deterministic ICER of £30,226/QALY and probability cost-effective 0.448.

The advantage of making all code open source is that very clever members of the community, in this case Dr. Sanchez Alvarez, can check your code for errors. The downside is that you occasionally need to make these embarrassing admissions.

The original article and the online supplementary information file have been corrected.

