# Correction: Deterministic and Probabilistic Analysis of a Simple Markov Model: How Different Could They Be?

**DOI:** 10.1007/s40258-023-00828-2

**Published:** 2023-09-27

**Authors:** Howard Thom

**Affiliations:** 1 Bristol Medical School: Population Health Sciences, University of Bristol, Bristol, UK; 2 Clifton Insight, Bristol, UK

**Correction: Applied Health Economics and Health Policy (2022) 20:447–449** 10.1007/s40258-021-00700-1

This article was originally published under a CC BY-NC 4.0 license, but has now been made available under a Creative Commons Attribution 4.0 International License (https://creativecommons.org/licenses/by/4.0/), which permits use, sharing, adaptation, distribution and reproduction in any medium or format, as long as you give appropriate credit to the original author(s) and the source, provide a link to the Creative Commons licence, and indicate if changes were made. The PDF and HTML versions of the paper have been modified accordingly.

The original article has been corrected.

