# Supplementary material for: Deterministic and Probabilistic Analysis of a Simple Markov Model: How Different Could They Be?
Source: Appl Health Econ Health Policy. 2022 Jan 20;20(3):447–9. doi: 10.1007/s40258-021-00700-1 (PMC9021067; doi:10.1007/s40258-021-00700-1)
Supplement: Supplementary file 1 — Supplementary file1 (DOCX 46 kb) [file 40258_2021_700_MOESM1_ESM.docx]

# Deterministic and Probabilistic Analysis of a Simple Markov Model: How different could they be?

Submitted to: Applied Health Economics and Health Policy

Howard Thom^1,2^

^1^Bristol Medical School: Population Health Sciences, University of Bristol, UK.

^2^Clifton Insight, Bristol, UK.

ORCID ID: <https://orcid.org/0000-0001-8576-5552>

Corresponding author email address: [howard.thom@bristol.ac.uk](mailto:howard.thom@bristol.ac.uk)

### Funding

This work was funded by the UK Medical Research Council grant MR/S036709/1.

### Conflicts of interest

I have received consulting fees from Novartis Pharma AG, Pfizer Inc, Roche Holding AG, Eisai Inc, Argenx, Janssen Pharmaceutical and BMS. None is directly related to this work.

# Supplementary materials

## A1. Further details on the simulation study

The Markov model used for simulation study is illustrated in Figure A1. Parameter values used for the general simulation study are presented in Table A1. They are only varied across scenarios for the probabilities of adverse event and death under treatment 1 and log odds ratios for treatment 2 relative to treatment 1. Costs and utilities are sampled from the same distribution across scenarios. The parameter values used in the two worse case scenarios are also provided in Table A1.

Figure A1 Diagram of 3-state Markov model used in simulation study


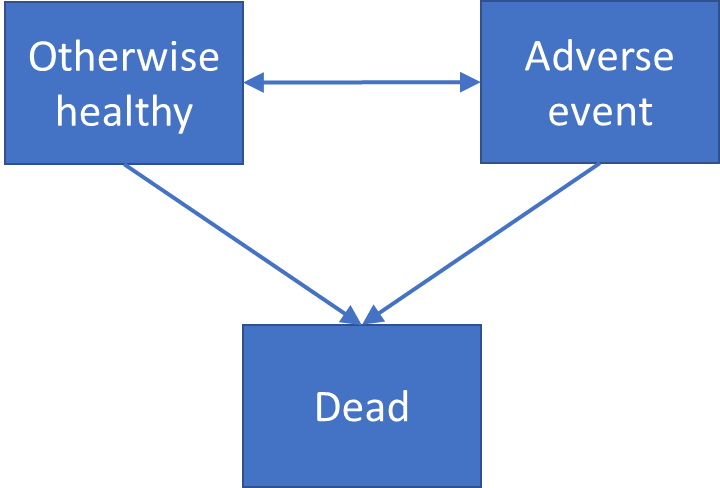


Table A1 Values of parameters used in simulation and in two worst case scenarios. One where ICER difference is maximised and another where ICERs disagree and CEAC of probabilistic analysis is maximised. Both are among scenarios where treatment 2 vs 1 incremental costs and effects are positive

| **Parameter** | **General simulation values** | | | **Values for scenarios of interest** | |
| --- | --- | --- | --- | --- | --- |
|  | **Distribution** | **Parameter 1** | **Parameter 2** | **Maximum ICER difference** | **Maximum CEAC inconsistency** |
| **Probability adverse event treatment 1** | Gamma(Shape, Scale) | Uniform(5, 50) | 100 | Gamma(6.864, 100.000) | Gamma(5.842, 100.000) |
| **Probability death treatment 1** | Gamma(Shape, Scale) | Uniform(5, 50) | 100 | Gamma(27.919, 100.000) | Gamma(6.449, 100.000) |
| **Log odds ratio adverse event treatment 2** | Normal(Mean, SD) | Uniform(0.1, 2.5) | 0.2 | Normal(0.657, sd = 0.200) | Normal(0.270, sd = 0.200) |
| **Log odds ratio death treatment 2** | Normal(Mean, SD) | Uniform(-1.5, -0.1) | 0.2 | Normal(-0.110, sd = 0.200) | Normal(-0.598, sd = 0.200) |
| **Utility healthy** | Normal(0.8, sd=0.05) | | | | |
| **Utility adverse event** | Normal(0.6, sd=0.1) | | | | |
| **Cost healthy** | Normal(1000, sd=100) | | | | |
| **Cost adverse event** | Normal(2000, sd=250) | | | | |
| **Cost treatment 1** | Normal(9000, sd=100) | | | | |
| **Cost treatment 2** | Normal(10000, sd=500) | | | | |

## A2. Further results on the simulation study

Table A2 Cross comparison of probabilistic and deterministic analyses across 10,000 simulation scenarios. Rows correspond to categories of conclusion under probabilistic analysis. Each row gives the proportion of these scenarios with four categories of conclusions under deterministic analysis. Diagonal elements indicate agreement between analyses.

|  | **Deterministic: Treatment 1 dominant** | **Deterministic: Treatment 1 cost-effective but not dominant** | **Deterministic: Treatment 2 cost-effective but not dominant** | **Deterministic: Treatment 2 dominant** |
| --- | --- | --- | --- | --- |
| **Probabilistic: Treatment 1 dominant** | 0.993 | 0.005 | 0.002 | 0.000 |
| **Probabilistic: Treatment 1 cost-effective but not dominant** | 0.004 | 0.980 | 0.016 | 0.000 |
| **Probabilistic: Treatment 2 cost-effective but not dominant** | 0.006 | 0.063 | 0.931 | 0.000 |
| **Probabilistic: Treatment 2 dominant*** | NA | NA | NA | NA |

*Treatment 2 was not dominant under any scenarios using either probabilistic or deterministic analysis

Table A3 Comparing deterministic and probabilistic results for two worst case scenarios. “Maximum ICER difference” where ICER difference is maximised. “Maximum CEAC inconsistency” where ICERs disagree, with deterministic ICER above £30,000/QALY, and the probability of treatment 2 being cost-effective at £20,000/QALY (i.e. the £20,000/QALY point of the CEAC) is maximised. Both are among scenarios where treatment 2 vs 1 incremental costs and effects are positive.*

| **Scenario** | **Maximum ICER difference** | **Maximum CEAC inconsistency** |
| --- | --- | --- |
| **Incremental costs deterministic (£)** | 1442.54 | 1944.96 |
| **Incremental effects deterministic (QALYs)** | 0.061 | 0.064 |
| **ICER deterministic (£/QALY)** | 23536.71 | 30226.74 |
| **Incremental net benefit deterministic at £20,000/QALY (£)** | -216.76 | -658.048 |
| **Incremental costs probabilistic (£)** | 1478.84 (51.85, 2968.26) | 1988.62 (403.28, 3727.22) |
| **Incremental effects probabilistic (£)** | 0.075 (-0.257, 0.434) | 0.075 (-0.268, 0.434) |
| **ICER probabilistic (£/QALY)** | 19660.67 | 26382.35 |
| **Incremental net benefit probabilistic at £20,000/QALY (£)** | 25.52 (-5775.73, 6302.08) | -481.08 (-6811.14, 5854.61) |
| **Probability of treatment 2 being cost-effective (i.e. CEAC) £20,000/QALY** | 0.478 | 0.448 |
| **EVPI (£)** | 776.36 | 839.60 |

*CEAC=Cost Effectiveness Acceptability Curve, EVPI=Expected Value of Perfect Information, ICER=Incremental Cost Effectiveness Ratio, QALY=Quality Adjusted Life Year

## A3. R code listing

Two scripts are provided. Run_markov_model.R is the main code for running both the probabilistic and deterministic Markov model. The main simulation study file is deterministic_vs_probabilistic.R, which calls the Markov model for each scenario of parameter values.

### run_markov_model.R

*# 3-state Markov model for simulation exploration comparing DSA and PSA*

*# Howard Thom 2020-21.*

*# With ackowledgement to Mi Jun Keng of Oxford University for optimization using lapply*

*# Model description:*

*# State 1 is healthy*

*# State 2 is adverse event*

*# State 3 is death*

*# Code runs both probabilistic and deterministic analysis*

*run_markov_model <- function(p_death_shape1 = 25,*

*p_death_shape2 = 200,*

*p_ae_shape1 = 10,*

*p_ae_shape2 = 200,*

*lor_death_mean = -0.5,*

*lor_death_sd = 0.1,*

*lor_ae_mean = 0.5,*

*lor_ae_sd = 0.1,*

*random_seed = 14143){*

*set.seed(random_seed)*

*# Define the number and names of treatments*

*# These are Standard of Care with website*

*# and Standard of Care without website*

*n_treatments <- 2*

*treatment_names <- c("Treatment 1", "Treatment 2")*

*# Define the number and names of states of the model*

*# Any number is allowed and states are named "State 1", "State 2", etc_*

*n_states <- 3*

*state_names <- paste("State", c(1:n_states))*

*# Define the number of cycles*

*# This is 10 as the time horizon is 5 years and cycle length is 6 months*

*# The code will work for any even n_cycles (need to change the discounting code if*

*# an odd number of cycles is desired)*

*n_cycles <- 10*

*# Define simulation parameters*

*# This is the number of PSA samples to use*

*n_samples <- 1000*

*#############################################################################*

*## Input parameters #########################################################*

*#############################################################################*

*# There is one transition matrix for each reatment option and each PSA sample*

*# Store them in an array with (before filling in below) NA entries*

*transition_matrices <- array(dim = c(n_treatments, n_samples, n_states, n_states),*

*dimnames = list(treatment_names, NULL, state_names, state_names))*

*# Now define the QALYS associated with the states per cycle*

*# There is one for each PSA sample and each state*

*# Store in an NA array and then fill in below*

*state_qalys <- array(dim = c(n_samples, n_states), dimnames = list(NULL, state_names))*

*# And finally define the state costs*

*# There is one for each PSA sample and each state*

*# Store in an NA array and then fill in below*

*state_costs <- array(dim = c(n_samples, n_states), dimnames = list(NULL, state_names))*

*# Treatment 2 reduces death but increases adverse event relative to treatment 1*

*probability_death <- probability_ae <- matrix(nrow = n_treatments, ncol = n_samples)*

*rownames(probability_death) <- rownames(probability_ae) <- treatment_names*

*probability_death[1, ] <- rbeta(n_samples, p_death_shape1, p_death_shape2)*

*probability_ae[1,] <- rbeta(n_samples, p_ae_shape1, p_ae_shape2)*

*lor_death <- rnorm(n_samples, lor_death_mean, lor_death_sd)*

*lor_ae <- rnorm(n_samples, lor_ae_mean, lor_ae_sd)*

*probability_death[2, ] <- expit(logit(probability_death[1, ]) + lor_death)*

*probability_ae[2, ] <- expit(logit(probability_ae[1, ]) + lor_ae)*

*# Transitions from state 1 (healthy)*

*transition_matrices["Treatment 1", , "State 1", "State 2"] <- probability_ae[1, ]*

*transition_matrices["Treatment 1", , "State 1", "State 3"] <- probability_death[1, ]*

*transition_matrices["Treatment 2", , "State 1", "State 2"] <- probability_ae[2, ]*

*transition_matrices["Treatment 2", , "State 1", "State 3"] <- probability_death[2, ]*

*# Ensure rows sum to 1*

*transition_matrices["Treatment 1", , "State 1", "State 1"] <- 1 - rowSums(transition_matrices["Treatment 1", , "State 1", c(2:3)])*

*transition_matrices["Treatment 2", , "State 1", "State 1"] <- 1 - rowSums(transition_matrices["Treatment 2", , "State 1", c(2:3)])*

*# Transitions from state 2 (adverse event)*

*# Patients recover unless they have the event again*

*transition_matrices["Treatment 1", , "State 2", "State 2"] <- probability_ae[1, ]*

*transition_matrices["Treatment 2", , "State 2", "State 2"] <- probability_ae[2, ]*

*# Death probability unaffected by adverse event*

*transition_matrices["Treatment 1", , "State 2", "State 3"] <- probability_death[1, ]*

*transition_matrices["Treatment 2", , "State 2", "State 3"] <- probability_death[2, ]*

*# Ensure rows sum to 1*

*transition_matrices["Treatment 1", , "State 2", "State 1"] <- 1 - rowSums(transition_matrices["Treatment 1", , "State 2", c(2:3)])*

*transition_matrices["Treatment 2", , "State 2", "State 1"] <- 1 - rowSums(transition_matrices["Treatment 2", , "State 2", c(2:3)])*

*# Transitions from state 3 (dead)*

*# Patients remain in death state*

*transition_matrices[, , "State 3", c("State 1", "State 2")] <- 0*

*transition_matrices[, , "State 3", "State 3"] <- 1*

*# Define state utilities and costs*

*for(i_state in 1:n_states)*

*{*

*# State utilities*

*# Healthy state is still diseased so imperfect*

*# Divide by 2 as cycle length is 6 months*

*state_qalys[,"State 1"] <- rnorm(n_samples, 0.8, 0.05) / 2*

*state_qalys[,"State 2"] <- rnorm(n_samples, 0.6, 0.1) / 2*

*state_qalys[,"State 3"] <- 0*

*# State costs*

*# Assumed normal with sd small enough to avoid negative values*

*state_costs[,"State 1"] <- rnorm(n_samples, mean = 1000, sd = 100)*

*# Adverse event has more severe costs*

*state_costs[,"State 2"] <- rnorm(n_samples, mean = 2000, sd = 250)*

*# Dead has no costs*

*state_costs[,"State 3"] <- 0*

*}*

*# Define the treatment costs*

*# One for each PSA sample and each treatment*

*treatment_costs <- array(dim = c(n_treatments, n_samples), dimnames = list(treatment_names, NULL))*

*# Treatment costs are random but treatment 1 is slightly cheaper and more uncertain*

*treatment_costs["Treatment 2", ] <- rnorm(n_samples, 10000, 500)*

*treatment_costs["Treatment 1", ] <- rnorm(n_samples, 9000, 100)*

*# Deterministic analysis*

*# Define deterministic parameters as average of probabilistic*

*transition_matrices_deterministic <- array(NA, dim = c(n_treatments, n_states, n_states),*

*dimnames = list(treatment_names, state_names, state_names))*

*transition_matrices_deterministic[1, , ] <- apply(transition_matrices[1, , , ], c(2, 3), mean)*

*transition_matrices_deterministic[2, , ] <- apply(transition_matrices[2, , , ], c(2, 3), mean)*

*state_qalys_deterministic <- colMeans(state_qalys)*

*state_costs_deterministic <- colMeans(state_costs)*

*treatment_costs_deterministic <- rowMeans(treatment_costs)*

*# Deterministic simulation*

*# Array of cohort vectors at each cycle for each treatment*

*cohort_vectors_deterministic <- array(NA, dim = c(n_treatments, n_cycles, n_states),*

*dimnames = list(treatment_names, NULL, state_names))*

*cohort_vectors_deterministic[, 1, "State 1"] <- 1*

*cohort_vectors_deterministic[, 1, paste("State", c(2:n_states))] <- 0*

*# Array of cycle costs and qalys*

*cycle_costs_deterministic <- array(dim = c(n_treatments, n_cycles),*

*dimnames = list(treatment_names, NULL))*

*cycle_qalys_deterministic <- array(dim = c(n_treatments, n_cycles),*

*dimnames = list(treatment_names, NULL))*

*# Objects to store deterministic results*

*total_costs_deterministic <- total_qalys_deterministic <- rep(NA, 2)*

*names(total_costs_deterministic) <- names(total_qalys_deterministic) <- treatment_names*

*# Vector used for discounting over 10 6-month cycles*

*disc_vec <- (1 / 1.035)^rep(c(0:(n_cycles / 2 - 1)), each = 2)*

*############################################################################*

*# Deterministic cohort simulation*

*############################################################################*

*for(i_treatment in 1:n_treatments) {*

*for(i_cycle in 2:n_cycles) {*

*# Update cohort vector using transition matrix*

*cohort_vectors_deterministic[i_treatment, i_cycle, ] <-*

*cohort_vectors_deterministic[i_treatment, i_cycle - 1, ] %*% transition_matrices_deterministic[i_treatment,,]*

*}*

*# Work out (undiscounted) cycle costs and qalys*

*cycle_costs_deterministic[i_treatment, ] <- cohort_vectors_deterministic[i_treatment, , ] %*% state_costs_deterministic*

*cycle_qalys_deterministic[i_treatment, ] <- cohort_vectors_deterministic[i_treatment, , ] %*% state_qalys_deterministic*

*# Discount and sum*

*total_costs_deterministic[i_treatment] <- cycle_costs_deterministic[i_treatment, ] %*% disc_vec*

*total_qalys_deterministic[i_treatment] <- cycle_qalys_deterministic[i_treatment, ] %*% disc_vec*

*}*

*# Add the treatment costs*

*total_costs_deterministic <- total_costs_deterministic + treatment_costs_deterministic*

*# Incremental costs and effects*

*incremental_costs_deterministic <- total_costs_deterministic[2] - total_costs_deterministic[1]*

*incremental_qalys_deterministic <- total_qalys_deterministic[2] - total_qalys_deterministic[1]*

*# ICER for deterministic decision making*

*icer_deterministic <- incremental_costs_deterministic / incremental_qalys_deterministic*

*############################################################################*

*# Probabilistic cohort simulation*

*############################################################################*

*# Build an array to store the cohort vector at each cycle*

*# Each cohort vector has n_states elements: probability of being in each state,*

*# There is one cohort vector for each treatment, for each PSA sample, for each cycle_*

*cohort_vectors <- array(dim = c(n_treatments, n_samples, n_cycles, n_states),*

*dimnames = list(treatment_names, NULL, NULL, state_names))*

*# Assume that everyone starts in first state*

*cohort_vectors[, , 1, "State 1"] <- 1*

*cohort_vectors[, , 1, paste("State", c(2:n_states))] <- 0*

*# Build an array to store the costs and QALYs accrued per cycle*

*# One for each treatment, for each PSA sample, for each cycle*

*# These will be filled in below in the main model code*

*# Then discounted and summed to contribute to total costs and total QALYs*

*cycle_costs <- array(dim = c(n_treatments, n_samples, n_cycles),*

*dimnames = list(treatment_names, NULL, NULL))*

*cycle_qalys <- array(dim = c(n_treatments, n_samples, n_cycles),*

*dimnames = list(treatment_names, NULL, NULL))*

*# Build arrays to store the total costs and total QALYs*

*# There is one for each treatment and each PSA sample*

*# These are filled in below using cycle_costs,*

*# treatment_costs, and cycle_qalys*

*total_costs <- array(dim = c(n_treatments, n_samples),*

*dimnames = list(treatment_names, NULL))*

*total_qalys <- array(dim = c(n_treatments, n_samples),*

*dimnames = list(treatment_names, NULL))*

*# The remainder of the cohort_vectors will be filled in by Markov updating below*

*lapply(c(1:n_treatments), function(i_treatment){*

*transition_matrices_tr <- transition_matrices[i_treatment, , , ]*

*cohort_vectors_tr <- cohort_vectors[i_treatment, , , ]*

*cycle_costs_tr <- cycle_costs[i_treatment, , ]*

*cycle_qalys_tr <- cycle_qalys[i_treatment, , ]*

*treatment_costs_tr <- treatment_costs[i_treatment, ]*

*total_costs_tr <- total_costs[i_treatment, ]*

*total_qalys_tr <- total_qalys[i_treatment, ]*

*# Loop over the PSA samples*

*for(i_sample in 1:n_samples)*

*{*

*transition_matrices_tr_sample <- transition_matrices_tr[i_sample, , ]*

*cohort_vectors_tr_sample <- cohort_vectors_tr[i_sample, , ]*

*# Loop over the cycles*

*# Cycle 1 is already defined so only need to update cycles 2:n_cycles*

*for(i_cycle in 2:n_cycles)*

*{*

*# Markov update*

*# Multiply previous cycle's cohort vector by transition matrix*

*# i_e_ pi_j = pi_(j-1)*P*

*cohort_vectors_tr_sample[i_cycle, ] <- cohort_vectors_tr_sample[i_cycle - 1, ] %*% transition_matrices_tr_sample*

*}*

*cycle_costs_tr[i_sample, ] <- cohort_vectors_tr_sample %*% state_costs[i_sample, ]*

*cycle_qalys_tr[i_sample, ] <- cohort_vectors_tr_sample %*% state_qalys[i_sample, ]*

*total_costs_tr[i_sample] <- treatment_costs_tr[i_sample] + cycle_costs_tr[i_sample,] %*% disc_vec*

*total_qalys_tr[i_sample] <- cycle_qalys_tr[i_sample,] %*% disc_vec*

*}*

*return(list(total_qalys = total_qalys_tr, total_costs = total_costs_tr))*

*}) -> output_list*

*names(output_list) <- treatment_names*

*#############################################################################*

*## Analysis of results ######################################################*

*#############################################################################*

*output <- list()*

*# Average costs*

*output$average_costs <- sapply(treatment_names, function(tx){mean(output_list[[tx]]$total_costs)})*

*# Average effects (in QALY units)*

*output$average_effects <- sapply(treatment_names, function(tx){mean(output_list[[tx]]$total_qalys)})*

*# Incremental costs and effects of treatment 2 relative to treatment 1*

*output$incremental_costs <- output_list[["Treatment 2"]]$total_costs - output_list[["Treatment 1"]]$total_costs*

*output$incremental_effects <- output_list[["Treatment 2"]]$total_qalys - output_list[["Treatment 1"]]$total_qalys*

*# For comparison of deterministic and probabilistic results*

*output$average_incremental_effects <- mean(output$incremental_effects)*

*output$average_incremental_costs <- mean(output$incremental_costs)*

*# The ICER comparing Standard of care with website to standard of care*

*# This is much lower than the £20,000 willingness-to-pay threshold indicating*

*# good value for money*

*output$ICER<-mean(output$incremental_costs) / mean(output$incremental_effects)*

*# Deterministic results for comparison*

*output$incremental_costs_deterministic <- incremental_costs_deterministic*

*output$incremental_qalys_deterministic <- incremental_qalys_deterministic*

*output$icer_deterministic <- icer_deterministic*

*output$incremental_net_benefit_deterministic <- 20000 * incremental_qalys_deterministic - incremental_costs_deterministic*

*# Incremental net benefit at the £20,000 willingness-to-pay*

*# Sometimes positive (website more cost-effective) and sometimes negative (SoC more cost-effective)*

*# Need to look at averages and consider probabilities of cost-effectiveness*

*output$incremental_net_benefit <- 20000 * output$incremental_effects - output$incremental_costs*

*# Average incremental net benefit*

*# This is positive indicating cost-effectiveness at the £20,000 threshold*

*output$average_inb <- mean(output$incremental_net_benefit)*

*# Probability cost-effective*

*# This is the proportion of samples for which the incremental net benefit is positive*

*output$probability_cost_effective <- sum(output$incremental_net_benefit > 0) / n_samples*

*# EVPI using probabilistic resutls (can't be estimated with deterministic)*

*# Expected max based on perfect info minus expected max based on current info*

*net_benefit <- matrix(nrow = n_samples, ncol = n_treatments)*

*net_benefit[, 1] <- output_list[["Treatment 1"]]$total_qalys * 20000 - output_list[["Treatment 1"]]$total_qalys*

*net_benefit[, 2] <- output_list[["Treatment 2"]]$total_qalys * 20000 - output_list[["Treatment 2"]]$total_qalys*

*output$evpi <- mean(apply(net_benefit, c(1), max)) - max(colMeans(net_benefit))*

*# Return results of this iteration of the simulation*

*return(output)*

*}*

### Deterministic_vs_probabilistic.R

*# Markov model comparing deterministic to probabilistic results*

*# Howard Thom 3-June-2020*

*library(ggplot2)*

*library(dplyr)*

*library(hrbrthemes)*

*# Set working directory*

*setwd("~/Bristol/PSA vs DSA Markov/Simulation exploration")*

*# Load the Markov model code*

*# Rstudio had a bug on 3-June-2020 that prevented the following line from working*

*source("run_markov_model.R")*

*## Utility functions ###############################################*

*# Logistic link function to convert probabilities to log odds scale*

*logit<-function(x)*

*{*

*return(log(x/(1-x)))*

*}*

*# Inverse of logit to convert log odds to probability scale*

*expit<-function(x)*

*{*

*return(1/(1+exp(-x)))*

*}*

*# Number of parameter values to explore*

*n_simulations <- 10000*

*# Results table*

*model_results_table <- matrix(NA, nrow = n_simulations, ncol = 15)*

*colnames(model_results_table) <- c("Scenario", "Incremental costs deterministic", "Incremental effects deterministic",*

*"ICER deterministic", "Incremental net benefit deterministic",*

*"Incremental costs probabilistic", "Incremental effects probabilistic",*

*"ICER probabilistic", "CEAC", "Dominance deterministic", "Dominance probabilistic",*

*"Incremental costs summary", "Incremental effects summary",*

*"Incremental net benefit", "EVPI")*

*model_results_table[, c(10,11)] <- "No dominance"*

*par_vector <- list()*

*# Function to format resutls*

*format_results <- function(x, n_digits = 3, med = FALSE) {*

*if(!med){ return(paste0(format(mean(x), digits = n_digits, nsmall = n_digits),*

*" (", format(quantile(x, probs = 0.025), digits = n_digits, nsmall = n_digits),*

*", ", format(quantile(x, probs = 0.975), digits = n_digits, nsmall = n_digits),*

*")"))*

*} else{*

*return(paste0(format(median(x), digits = n_digits, nsmall = n_digits),*

*" (", format(quantile(x, probs = 0.025), digits = n_digits, nsmall = n_digits),*

*", ", format(quantile(x, probs = 0.975), digits = n_digits, nsmall = n_digits),*

*")"))*

*}*

*}*

*# Function to format the parameter vector for easy comparison*

*# Note that AE and death are reversed in order*

*format_parameter_vector <- function(x, n_digits = 3) {*

*formatted_vector <- rep(NA, 4)*

*names(formatted_vector) <- c("Probability AE treatment 1",*

*"Probability Death treatment 1",*

*"LOR AE treatment 2",*

*"LOR Death treatment 2")*

*formatted_vector[1] <- paste0("Gamma(", format(x[3], digits = n_digits, nsmall = n_digits), ", ", format(x[4], digits = n_digits, nsmall = n_digits), ")")*

*formatted_vector[2] <- paste0("Gamma(", format(x[1], digits = n_digits, nsmall = n_digits), ", ", format(x[2], digits = n_digits, nsmall = n_digits), ")")*

*formatted_vector[3] <- paste0("Normal(", format(x[7], digits = n_digits, nsmall = n_digits), ", sd = ", format(x[8], digits = n_digits, nsmall = n_digits), ")")*

*formatted_vector[4] <- paste0("Normal(", format(x[5], digits = n_digits, nsmall = n_digits), ", sd = ", format(x[6], digits = n_digits, nsmall = n_digits), ")")*

*return(formatted_vector)*

*}*

*# Use a general seed to generate random numbers*

*set.seed(14523451)*

*# But a different seed for each model simulation, to ensure good variation in*

*# simulated input parameters*

*random_seeds <- ceiling(runif(n_simulations, 0, 10000000))*

*for(i_sim in 1:n_simulations) {*

*# Simulate model parameters*

*par_vector[[i_sim]] <- c(runif(1, 5, 50), 100, runif(1, 5, 25), 100,*

*runif(1, -1.5, -0.1), 0.2, runif(1, 0.1, 2.5), 0.2)*

*model_output <- run_markov_model(p_death_shape1 = par_vector[[i_sim]][1],*

*p_death_shape2 = par_vector[[i_sim]][2],*

*p_ae_shape1 = par_vector[[i_sim]][3],*

*p_ae_shape2 = par_vector[[i_sim]][4],*

*lor_death_mean = par_vector[[i_sim]][5],*

*lor_death_sd = par_vector[[i_sim]][6],*

*lor_ae_mean = par_vector[[i_sim]][7],*

*lor_ae_sd = par_vector[[i_sim]][8],*

*random_seed = random_seeds[i_sim])*

*print(i_sim)*

*model_results_table[i_sim, -c(10:11)] <- c(paste("Scenario", i_sim),*

*model_output$incremental_costs_deterministic,*

*model_output$incremental_qalys_deterministic,*

*model_output$icer_deterministic,*

*model_output$incremental_net_benefit_deterministic,*

*model_output$average_incremental_costs,*

*model_output$average_incremental_effects,*

*model_output$ICER,*

*model_output$probability_cost_effective,*

*format_results(model_output$incremental_costs),*

*format_results(model_output$incremental_effects),*

*format_results(model_output$incremental_net_benefit),*

*model_output$evpi)*

*}*

*# Name the elements of list of parameter vectors*

*length(par_vector)*

*names(par_vector) <- paste("Scenario", c(1:n_simulations))*

*# Check if any treatment is dominant*

*model_results_table[ model_results_table[, "Incremental costs probabilistic"] > 0 & model_results_table[, "Incremental effects probabilistic"] < 0, "Dominance probabilistic"] <- "Treatment 1 dominant"*

*model_results_table[ model_results_table[, "Incremental costs probabilistic"] < 0 & model_results_table[, "Incremental effects probabilistic"] > 0, "Dominance probabilistic"] <- "Treatment 2 dominant"*

*model_results_table[ model_results_table[, "Incremental costs deterministic"] > 0 & model_results_table[, "Incremental effects deterministic"] < 0, "Dominance deterministic"] <- "Treatment 1 dominant"*

*model_results_table[ model_results_table[, "Incremental costs deterministic"] < 0 & model_results_table[, "Incremental effects deterministic"] > 0, "Dominance deterministic"] <- "Treatment 2 dominant"*

*# Save the simulations*

*save(model_results_table, par_vector, random_seeds, file = paste0("deterministic_vs_probabilistic_results_2", n_simulations, "_rda"))*

*#load(paste0("deterministic_vs_probabilistic_results_2", n_simulations, "_rda"))*

*###########################################################################*

*## Cross comparison of results ############################################*

*###########################################################################*

*cross_comparison <- matrix(NA, nrow = 4, ncol = 4)*

*colnames(cross_comparison) <- paste("Deterministic:",c("Treatment 1 dominant", "Treatment 1 CE",*

*"Treatment 2 CE", "Treatment 2 dominant"))*

*rownames(cross_comparison) <- paste("Probabilistic:",c("Treatment 1 dominant", "Treatment 1 CE",*

*"Treatment 2 CE", "Treatment 2 dominant"))*

*# Categories of conclusion using probabilistic results*

*treatment1_dominant_prob <- model_results_table[, "Dominance probabilistic"] == "Treatment 1 dominant"*

*treatment2_dominant_prob <- model_results_table[, "Dominance probabilistic"] == "Treatment 2 dominant"*

*# ICER only meaningful if inc costs and effects have same sign*

*icer_meaningful_prob <- (model_results_table[, "Incremental costs probabilistic"] >= 0 &*

*model_results_table[, "Incremental effects probabilistic"] >= 0) | (model_results_table[, "Incremental costs probabilistic"] <= 0 &*

*model_results_table[, "Incremental effects probabilistic"] <= 0)*

*treatment1_ce_prob <- model_results_table[, "ICER probabilistic"] >= 20000 & icer_meaningful_prob*

*treatment2_ce_prob <- model_results_table[, "ICER probabilistic"] < 20000 & icer_meaningful_prob*

*# Categories of conclusion using deterministic results*

*treatment1_dominant_det <- model_results_table[, "Dominance deterministic"] == "Treatment 1 dominant"*

*treatment2_dominant_det <- model_results_table[, "Dominance deterministic"] == "Treatment 2 dominant"*

*icer_meaningful_det <- (model_results_table[, "Incremental costs deterministic"] >= 0 &*

*model_results_table[, "Incremental effects deterministic"] >= 0) | (model_results_table[, "Incremental costs deterministic"] <= 0 &*

*model_results_table[, "Incremental effects deterministic"] <= 0)*

*treatment1_ce_det <- model_results_table[, "ICER deterministic"] >= 20000 & icer_meaningful_det*

*treatment2_ce_det <- model_results_table[, "ICER deterministic"] < 20000 & icer_meaningful_det*

*cross_comparison["Probabilistic: Treatment 1 dominant", ] <- c(*

*sum(treatment1_dominant_prob & treatment1_dominant_det),*

*sum(treatment1_dominant_prob & treatment1_ce_det),*

*sum(treatment1_dominant_prob & treatment2_ce_det),*

*sum(treatment1_dominant_prob & treatment2_dominant_det)) / sum(treatment1_dominant_prob)*

*cross_comparison["Probabilistic: Treatment 1 CE", ] <- c(*

*sum(treatment1_ce_prob & treatment1_dominant_det),*

*sum(treatment1_ce_prob & treatment1_ce_det),*

*sum(treatment1_ce_prob & treatment2_ce_det),*

*sum(treatment1_ce_prob & treatment2_dominant_det)) / sum(treatment1_ce_prob)*

*cross_comparison["Probabilistic: Treatment 2 dominant", ] <- c(*

*sum(treatment2_dominant_prob & treatment1_dominant_det),*

*sum(treatment2_dominant_prob & treatment1_ce_det),*

*sum(treatment2_dominant_prob & treatment2_ce_det),*

*sum(treatment2_dominant_prob & treatment2_dominant_det)) / sum(treatment2_dominant_prob)*

*cross_comparison["Probabilistic: Treatment 2 CE", ] <- c(*

*sum(treatment2_ce_prob & treatment1_dominant_det),*

*sum(treatment2_ce_prob & treatment1_ce_det),*

*sum(treatment2_ce_prob & treatment2_ce_det),*

*sum(treatment2_ce_prob & treatment2_dominant_det)) / sum(treatment2_ce_prob)*

*write.csv(cross_comparison, file = paste0("results/cross_comparison_", n_simulations, ".csv"))*

*###########################################################################*

*## Identifying extreme cases ##############################################*

*###########################################################################*

*# Search cases where incremental results are all positive (higher costs but higher effects)*

*# and where at least one ICER is below 20000*

*# for greatest difference between ICERs*

*positive_results_index <- rowSums(model_results_table[, c(2, 3, 6, 7)] < 0) == 0*

*atleast_one_icer_ce <- as.numeric(model_results_table[, "ICER probabilistic"]) < 20000 | as.numeric(model_results_table[, "ICER deterministic"]) < 20000*

*# Maximum difference between probabilistic and deterministic ICER*

*# among cases where the deterministic and probabilistic incremental costs and effects are positive (i.e. NE quadrant)*

*max_icer_difference <- (model_results_table[positive_results_index & atleast_one_icer_ce, ][which.max(abs(*

*as.numeric(model_results_table[positive_results_index & atleast_one_icer_ce, "ICER probabilistic"]) -*

*as.numeric(model_results_table[positive_results_index & atleast_one_icer_ce, "ICER deterministic"]) )), ])*

*write.csv(format_parameter_vector(par_vector[[max_icer_difference["Scenario"]]]),*

*file = paste("results/max_icer_difference_", n_simulations, "_parameters.csv"))*

*write.csv(max_icer_difference, file = paste("results/max_icer_difference_", n_simulations, ".csv"))*

*#Also look for case where the above is true but CEAC is maximised_*

*# Identify case where ICER is not cost-effective but CEAC is maximised*

*# among cases where the deterministic and probabilistic incremental costs and effects are positive (i.e. NE quadrant)*

*deterministic_icer_nce <- as.numeric(model_results_table[, "ICER deterministic"]) > 30000*

*max_ceac_inconsistency <- (model_results_table[positive_results_index & deterministic_icer_nce, ][which.max(*

*model_results_table[positive_results_index & deterministic_icer_nce, "CEAC"]), ])*

*write.csv(format_parameter_vector(par_vector[[max_ceac_inconsistency["Scenario"]]]),*

*file = paste("results/max_ceac_inconsistency_", n_simulations, "_parameters.csv"))*

*write.csv(max_ceac_inconsistency, file = paste("results/max_ceac_inconsistency_", n_simulations, ".csv"))*

*# Extreme case where deterministic says treatment 2 dominated but*

*# probabilistic says it is cost-effective*

*# Identify case where probabilistic ICER is minimised*

*# No such cases*

*treatment1_dominant_but_treatment2_ce <- which(model_results_table[, "Dominance deterministic"] == "Treatment 1 dominant" &*

*model_results_table[, "Dominance probabilistic"] != "Treatment 1 dominant" &*

*as.numeric(model_results_table[, "ICER probabilistic"]) < 20000)*

*min_temp <- which.min(as.numeric(model_results_table[treatment1_dominant_but_treatment2_ce, "ICER probabilistic"]))*

*dominant_but_ce <- model_results_table[treatment1_dominant_but_treatment2_ce[min_temp], ]*

*###########################################################################*

*## Plotting results #######################################################*

*###########################################################################*

*inb_probabilistic <- 20000 * as.numeric(model_results_table[, "Incremental effects probabilistic"]) -*

*as.numeric(model_results_table[, "Incremental costs probabilistic"])*

*inb_deterministic <- as.numeric(model_results_table[, "Incremental net benefit deterministic"])*

*format_results(inb_deterministic)*

*format_results(inb_probabilistic)*

*format_results(inb_probabilistic - inb_deterministic)*

*# Compare the distributions*

*inb_comparison <- data.frame(*

*type = c(rep("INB Deterministic", length(inb_deterministic)),*

*rep("INB Probabilistic", length(inb_probabilistic))),*

*value = c(inb_deterministic, inb_probabilistic)*

*)*

*# Represent it*

*p <- inb_comparison %>%*

*ggplot( aes(x=value, fill=type)) +*

*geom_histogram( color="#e9ecef", alpha=0.6, position = 'identity') +*

*labs(x = "£GBP",*

*y = "Counts") +*

*scale_fill_manual(values=c("#69b3a2", "#404080")) +*

*theme_ipsum() +*

*labs(fill="")*

*tiff(file = paste0("results/hist_inb_comparison_", n_simulations, ".tiff"))*

*plot(p)*

*dev.off()*
